# Using discrete choice experiments to define patient preferences for outcomes in trials

**DOI:** 10.1186/1745-6215-14-S1-O33

**Published:** 2013-11-29

**Authors:** Emily Fargher, Dyfrig Hughes, Adele Ring, Ann Jacoby, Margaret Rawnsley, Anthony Marson

**Affiliations:** 1 Bangor University, Bangor, UK; 2 University of Liverpool, Liverpool, UK; 3 Epilepsy Action, Leeds, UK; 4 Walton Centre for Neurology and Neurosurgery, Liverpool, UK

## Aims

(i) to identify outcomes of anti-epileptic drug (AED) treatment patients consider important; (ii) to elicit preferences for these outcomes and definitions of equivalence; (iii) to investigate if perceptions of acceptable trade-offs between benefits and harms differ across different subgroups.

## Methods

Web-based survey, containing discrete choice experiments (DCEs) to elicit preferences of three pre-defined groups of adults with epilepsy (n=750): (i) early epilepsy, (ii) established epilepsy, (iii) women of childbearing age (WOCBA). The DCEs contains five attributes, with two levels, defined using: semi-structured interviews with patients (n=56), a focus group with AED prescribers (n=8), and trial data. Each used the same fractional factorial design, folded into eight binary choices: Which medication would you prefer to take? Target sample size is 750 respondents, recruited via the Epilepsy Action website. Data will be analysed in STATA using a random effects logit model.

## Outcomes to date

Identified four common attributes: seizure-free, fewer seizures, depression and memory problems. Fifth attributes were feelings of aggression (early and established) and foetal harm (WOCBA). The qualitative findings suggest that preferences for negative outcomes vary by sub-group. The results of the discrete choice experiment will provide further information on acceptable trade-offs between the benefits of remission and fewer seizures, with potential harms; and thus indicate important outcomes for clinical trials.

## Conclusion

Exploring what people with epilepsy consider important for measuring AED effectiveness will ensure clinical services are focus on patient-defined needs and that future research is designed to assess appropriate patient-defined outcomes.

